# Design, synthesis and antimicrobial evaluation of pyrimidin-2-ol/thiol/amine analogues

**DOI:** 10.1186/s13065-017-0284-2

**Published:** 2017-06-09

**Authors:** Sangeeta Narwal, Sanjiv Kumar, Prabhakar Kumar Verma

**Affiliations:** Department of Pharmaceutical Sciences, Maharshi Dayanand University, Rohtak, Haryana 124001 India

**Keywords:** Pyrimidine derivatives, Antibacterial activity, Antifungal activity

## Abstract

**Background:**

Pyrimidine is an aromatic heterocyclic moiety containing nitrogen atom at 1st and 3rd positions and play an important role to forms the central core for different necessity of biological active compounds, from this facts, we have designed and synthesized a new class of pyrimidin-2-ol/thiol/amine derivatives and screened for its in vitro antimicrobial activity.

**Results and discussion:**

The synthesized pyrimidine derivatives were confirmed by IR, ^1^H/^13^C-NMR, Mass spectral studies and evaluated for their in vitro antimicrobial potential against Gram positive (*S. aureus* and *B. subtilis*), Gram negative (*E. coli, P. aeruginosa* and *S. enterica*) bacterial strains and fungal strain (*C. albicans* and *A. niger*) by tube dilution method and recorded minimum inhibitory concentration in µM/ml. The MBC and MFC values represent the lowest concentration of compound that produces in the range of 96–98% end point reduction of the used test bacterial and fungal species.

**Conclusion:**

In general all synthesized derivatives exhibited good antimicrobial activity. Among them, compounds **2, 5, 10, 11** and **12** have significant antimicrobial activity against used bacterial and fungal strains and also found to be more active than the standard drugs.

**Graphical abstract:**
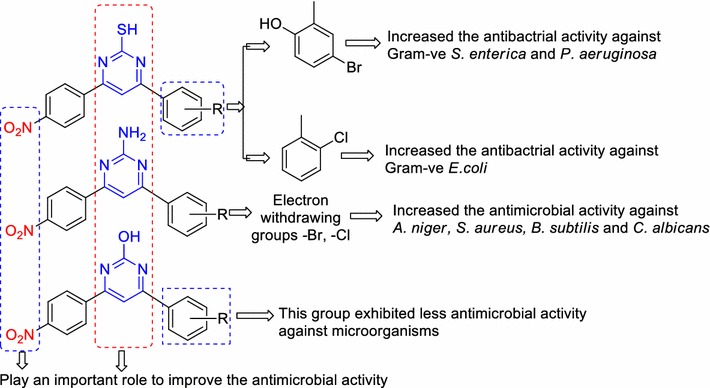
Pyrimidine is an aromatic heterocyclic moiety containing nitrogen atom at 1st and 3rd positions and play an important role to forms the central core for different necessity of biological active compounds, from this facts, we have designed and synthesized a new class of pyrimidin-2-ol/thiol/amine derivatives and screened for its in vitro antimicrobial activity. The synthesized pyrimidine derivatives were confirmed by IR, 1H/13C-NMR, Mass spectral studies and evaluated for their in vitro antimicrobial potential against Gram positive (*S. aureus* and* B. subtilis*), Gram negative (*E. coli*,* P. aeruginosa* and* S. enterica*) bacterial strains and fungal strain (*C. albicans* and* A. niger*) by tube dilution method and recorded minimum inhibitory concentration in µM/ml. The MBC and MFC values represent the lowest concentration of compound that produces in the range of 96–98% end point reduction of the used test bacterial and fungal species.

## Background

Antimicrobial agents are one of the most important weapons in the resistance of infection caused by bacterial strains. In the past few years, increase the resistance of microorganisms toward antimicrobial agents become a serious health problem so there is a need of safe, potent and novel antimicrobial agents [1]. Pyrimidine aromatic heterocyclic moiety containing nitrogen atom at 1st and 3rd positions and play an important role to forms the central core for different necessity of biological active compounds [2]. Pyrimidine is the structural unit of DNA and RNA which play an imperative role in various existence progressions. Pyrimidines are present among the three isomeric diazines. Most abundant pyrimidine is uracil, cytosine and thymine [3]. These derivatives are also known as *m*-diazine or 1,3-diazone can be regarded as cyclic amine and shows the various biological activities i.e. antiviral [4, 5]; anticancer [6]; antimicrobial [7]; anti-inflammatory [8]; analgesic [9]; antioxidant [10]; antimalarial [11].

Pyrimidine is used as parent substance for the synthesis of a wide variety of heterocyclic compounds and raw material for the synthesis of new molecule [12]. Pyrimidine ring complexes with different heterocyclic moiety found to be an essential part of natural products agrochemicals and veterinary products. A large measure of antimicrobial drugs such as ciprofloxacin, chloramphenicol, griseofulvin and nystatin are available for bacterial and fungal infections [13].

**Fig. 1:**
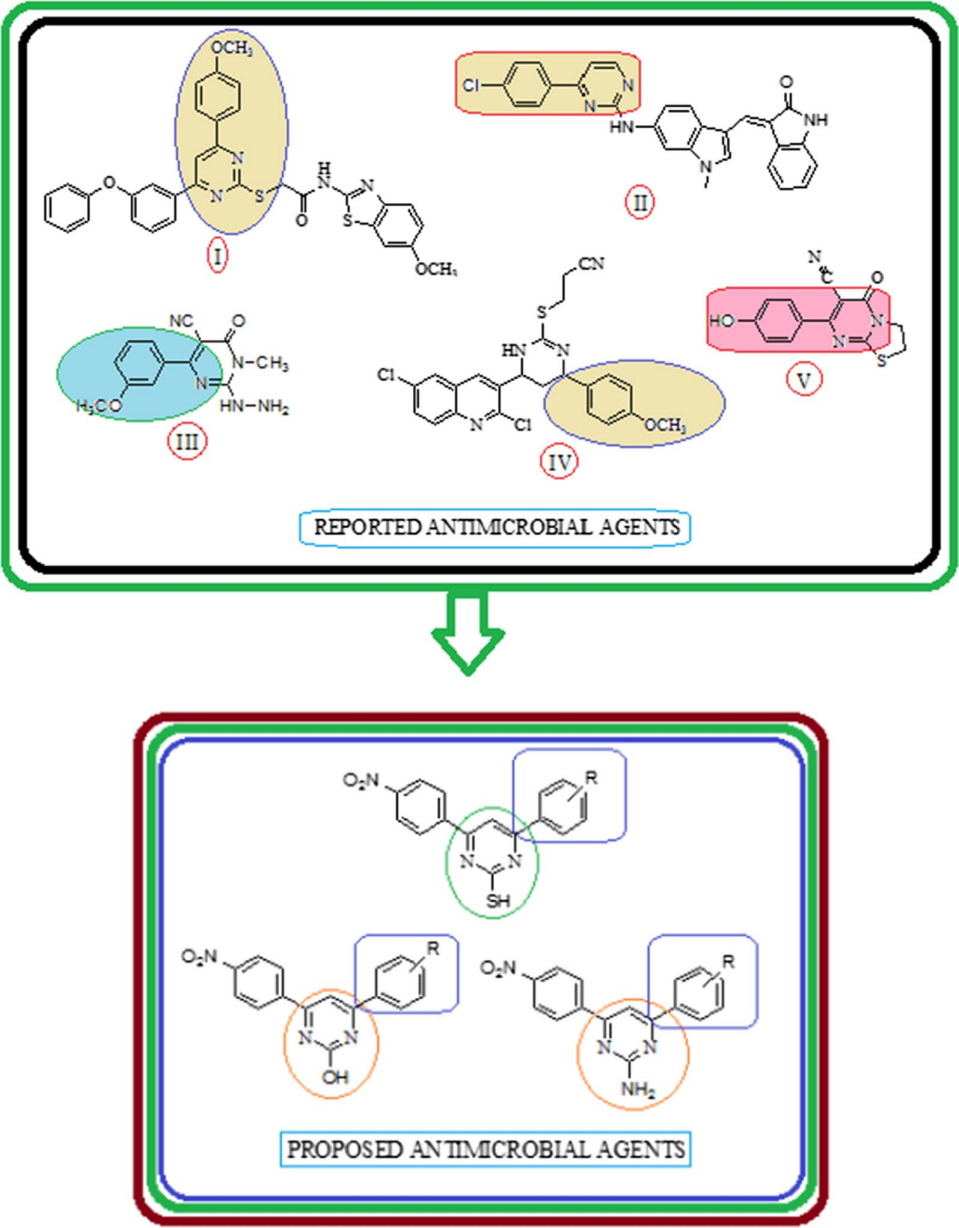
Design of proposed pyrimidine derivatives based on literature survey

**Scheme 1:**
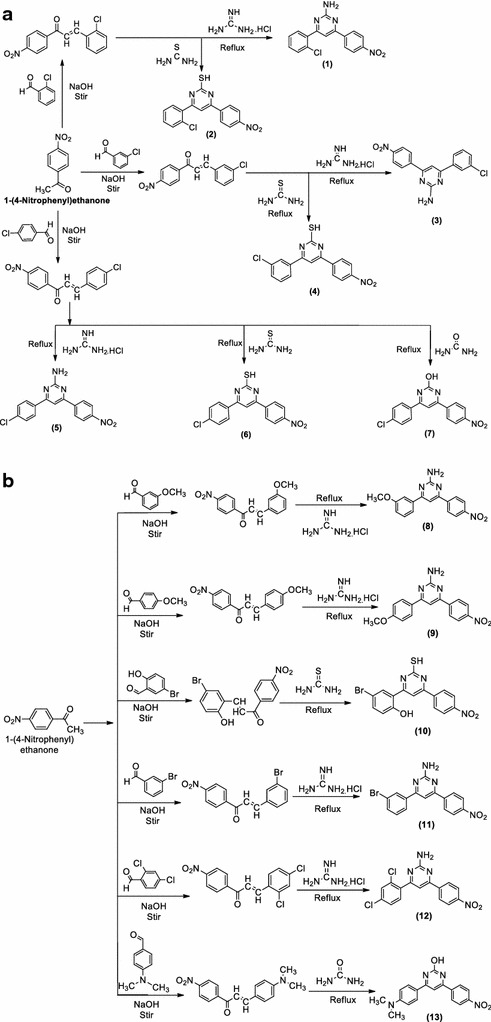
**a**, **b** Synthesis of 4-(substituted phenyl)-6-(4-nitrophenyl)pyrimidin-2-ol/thiol/amine derivatives

Recently, it was reported that *p*-methoxyphenyl group present on pyrimidine nucleus improved the antimicrobial activity of the pyrimidine derivative (**I**) [13], *p*-Chloro phenyl group present on pyrimidine nucleus [14] improved the anticancer activity of the pyrimidine derivatives **(II)**, *p*-Methoxyphenyl group present on pyrimidine derivatives **(III)** improved the antioxidant [15], *p*-Methoxyphenyl group present on pyrimidine ring **(IV)** improved the antitubercular activity of the pyrimidine derivatives [16], *p*-Hydroxy group present on pyrimidine nucleus **(V)** improved the antimicrobial of the pyrimidine compound [10]. The electron releasing (–OH and –OCH_3_) and electron withdrawing (–Cl) groups are present on different position of pyrimidine nucleus (**I, II, III**, **IV** and **V**) enhanced the biological activity of the pyrimidine derivatives, from this facts we developed a design of reported biological active agents and proposed antimicrobial agent which is presented in Fig. 1. In light of abovementioned facts, we hereby report to design, synthesis and antimicrobial screening of 4-(substituted phenyl)-6-(4-nitrophenyl) pyrimidin-2-ol/thiol/amine derivatives (Scheme 1a, b).

## Results and discussion

### Chemistry

Synthesis of pyrimidine derivatives (**1**–**13**) followed the general procedure discussed in synthetic Scheme 1a, b. The reaction of substituted chalcone in the presence of guanidine hydrochloride/urea/thiourea in methanolic solvent resulted in the formation of the final compounds. The physicochemical properties of newly synthesized compounds are presented in Table 1. The molecular structures of the synthesized compounds **(1**–**13)** were confirmed by FT-IR (KBr pellets, cm^−1^) and ^1^H/^13^C-NMR (CDCl_3_, δ ppm) spectral and elemental studies. The appearance of IR absorption band at 1404 cm^−1^ in the spectral data of synthesized derivatives **(1**–**13)** displayed the presence of Ar–OH (C–O str. and O–H in plane bend. vib.) category on the aromatic ring. The IR absorption band in the scale of 645–623 cm^−1^ corresponds to the C–Br stretching of aromatic-bromo compounds (**10** and **11)**. The existence of Ar–NO_2_ group asymmetric Ar–NO_2_ stretches in the scale of 1550–1510 cm^−1^. The existence of an arylalkyl ether category (Ar–OCH_3_) in compounds **8** and **9** are established by the existence of an IR absorption band around 2842–2829 cm^−1^. Halogen group in compounds **1**–**7** and **12** is indicated by the existence of Ar–Cl stretching vibrations at 732–848 cm^−1^. The impression of IR stretching at 2602–2627 and 623–709 cm^−1^ in the spectral data of synthesized compounds specified the existence of S–H and C–S group respectively. The appearance of IR stretching at 3379–3349 cm^−1^ spectral data of synthesized compounds specified the existence of –NH_2_ group. The impression of IR stretching vibration at 3100–3000 and 1580–1600 cm^−1^ in the spectral data of synthesized compounds specified the existence of C–H and C=C group respectively. The appearance of IR stretching 1670–1709 cm^−1^ in the spectral data of all synthesized compounds specified the existence of C=N group. The multiplet signals between 6.33 and 8.34 δ ppm in ^1^H-NMR spectra is indicative of aromatic proton of synthesized derivatives. The compounds **8** and **9** showed singlet at 3.01–3.34 δ ppm due to the existence of OCH_3_ of Ar–OCH_3_. All compounds showed singlet at 7.51–8.43 and 6.85–841 δ ppm due to the existence of N=CH and –CH groups in pyrimidine ring respectively. Compound **13** showed singlet at 2.19 δ ppm due to existence of –N(CH_3_)_2_ at the *para* position. Compounds, **1, 3, 5, 8, 11** and **12** showed singlet at 4.0–4.3 δ ppm due to existence of –NH_2_ at the *para* position and **2, 4, 6** and **10** showed singlet at 3.01–3.34 δ ppm due to existence of –SH group at the *para* position of the pyrimidine ring. The elemental screened studies of the 4-(substituted phenyl)-6-(4-nitrophenyl)pyrimidin-2-ol/thiol/amine were found within ± 0.39% of the theoretical results.

**Table 1:** The physicochemical properties of synthesized 4-(substituted phenyl)-6-(4-nitrophenyl) pyrimidin-2-ol/thiol/amine derivatives

Compounds	M. formula	M. weight	M.pt. (°C)	R_*f*_ value^a^	% Yield
Physicochemical properties					
**1.**	C_16_H_11_ClN_4_O_2_	326	80–82	0.45	75.00
**2.**	C_16_H_10_ClN_3_O_2_S	343	61–63	0.57	84.72
**3.**	C_16_H_11_ClN_4_O_2_	326	76–78	0.60	78.78
**4.**	C_16_H_10_ClN_3_O_2_S	343	90–92	0.62	72.54
**5.**	C_16_H_11_ClN_4_O_2_	326	122–124	0.58	82.22
**6.**	C_16_H_10_ClN_3_O_2_S	343	63–65	0.56	75.00
**7.**	C_16_H_10_ClN_3_O_3_	327	127–129	0.60	84.72
**8.**	C_17_H_14_N_4_O_3_	322	66–68	0.51	73.43
**9.**	C_17_H_14_N_4_O_3_	322	89–91	0.56	76.47
**10.**	C_16_H_10_BrN_3_O_3_S	404	59–61	0.61	81.81
**11.**	C_16_H_11_BrN_4_O_2_	371	153–155	0.41	64.00
**12.**	C_16_H_10_ClN_4_O_2_	361	87–89	0.42	87.61
**13.**	C_18_H_16_N_4_O_3_	336	156–158	0.45	77.38

^**a**^TLC mobile phase-benzene

#### In vitro antimicrobial activity

All the newly synthesized pyrimidine derivatives were examined for their in vitro antimicrobial activity against Gram positive *S. aureus* (MTCC 3160), *B. subtilis* (MTCC 441), Gram negative species: *E. coli* (MTCC 443), *P. aeruginosa* (MTCC 3542), *S. enteric* (MTCC 1165) and fungus species: *A. niger* (MTCC 281) and *C. albicans* (MTCC 227) strain using tube dilution method [17]. Dilutions of test and standard compounds were prepared in double strength nutrient broth for bacterial strains and sabouraud dextrose broth for fungal strains [18]. The minimum inhibitory concentration (MIC i.e. lowest concentration required of test substance to complete growth inhibition) values of standard drugs and synthesized compounds are presented in Table 2. From the results of antimicrobial evaluation it was observed that the entire synthesized compounds showed appreciable antimicrobial activity and different compounds were found to be active against different microorganisms. In case of Gram positive bacteria, compounds **12** (MIC_sa_ = 0.87 µM/ml) showed significant activity against *S. aureus* and **5** (MIC_bs_ = 0.96 µM/ml) exhibited most potent antibacterial activity against *B. subtilis*. In case of Gram negative bacteria, compounds **10** (MIC_se_ = 1.55 µM/ml) showed significant activity against *Salmonella enteric,* **2** (MIC_ec_ = 0.91 µM/ml) displayed more potent antibacterial activity against *E. coli* and **10** (MIC_pa_ = 0.77 µM/ml) exhibited most potent antibacterial activity against *P. aeruginosa*. Compound **12** (MIC_ca_ = 1.73 µM/ml) showed significant activity against *C. albicans* and **11** (MIC_an_ = 1.68 µM/ml) was found to be most potent antifungal agent against *A. niger*. All synthesized compounds having more antimicrobial potential than the standard cefadroxil (antibacterial) and fluconazole (antifungal) drugs and these compounds may be used as lead for the further discovery of new antimicrobial agents.

**Table 2:** Antimicrobial activity (MIC = µM/ml) of synthesized 4-(substituted phenyl)-6-(4-nitrophenyl) pyrimidin-2-ol/thiol/amine derivatives

Compounds no.	Minimum inhibitory concentration (MIC = µM/ml)						
	Bacterial strains					Fungal strains	
	Gram positive		Gram negative				
	*S. aureus* (MTCC 3160)	*B. subtilis* (MTCC 441)	*E. coli* (MTCC 443)	*P. aeruginosae* (MTCC 3542)	*S. enteric* (MTCC 1165)	*C. albicans* (MTCC 227)	*A. Niger* (MTCC 281)
**1.**	1.91	3.83	1.91	1.91	1.91	3.83	3.83
**2.**	1.82	3.64	0.91	1.82	1.82	1.82	3.64
**3.**	1.91	3.83	0.96	1.91	3.83	3.83	3.83
**4.**	3.64	3.64	1.82	0.91	3.64	3.64	3.64
**5.**	1.91	0.96	1.91	1.91	3.83	1.91	3.83
**6.**	1.82	3.64	1.82	1.82	3.64	1.82	3.64
**7.**	3.81	3.81	1.91	1.91	3.81	1.91	3.81
**8.**	3.88	3.88	1.94	3.88	3.88	1.94	3.88
**9.**	1.94	3.88	1.94	3.88	3.88	3.88	3.88
**10.**	3.09	1.55	1.55	0.77	1.55	3.09	3.09
**11.**	1.68	3.37	1.68	3.37	3.37	3.37	1.68
**12.**	0.87	1.73	1.73	1.73	1.73	1.73	3.46
**13.**	0.93	3.72	1.86	3.72	3.72	1.86	3.72
DMSO	0.00	0.00	0.00	0.00	0.00	0.00	0.00
Cefadroxil	1.72	1.72	1.72	1.72	1.72	–	–
Fluconazole	–	–	–	–	–	2.04	2.04

#### Determination of MBC/MFC

After recorded the MIC results of the synthesized compounds in concentration of (50, 25, 12.5, 6.25, 3.125, 1.56) µM/ml against microbial species i.e. Gram positive bacteria (*S. aureus* and *B. subtilis*), Gram negative bacteria (*E. coli, P. aeruginosa* and *S. enterica*) and fungal strain (*C. albicans* and *A. niger*) then their minimum bactericidal concentration (MBC) and fungicidal concentration (MFC) were determined by petri dish method using nutrient agar media (antibacterial) and sabouraud dextrose agar media (antifungal) by subculturing 100 μl of culture from each test tube that remained clear in the MIC determination into fresh medium. The MBC and MFC values represent the lowest concentration of compound that produces in the range of 96–98% end point reduction of the used test bacterial and fungal species [19].

### SAR (structure activity relationship) studies

**Fig. 2:**
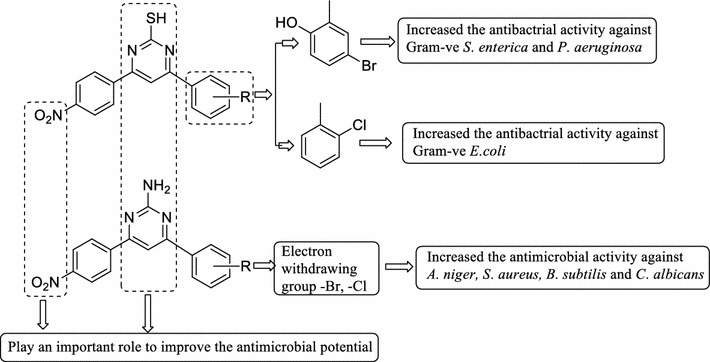
Structural requirements for the antimicrobial activity of the synthesized derivatives

From the antimicrobial testing results of synthesized 4-(substituted phenyl)-6-(4-nitrophenyl)pyrimidin-2-ol/thiol/amine derivatives, the subsequent structure activity relationship can be derived in Fig. 2.

- Presence of electron withdrawing group (–Cl, Compounds **2, 5** and **12**) on benzylidene portion improved the antimicrobial activity of the synthesized compounds against *S. aureus, E. coli, B. subtilis* and *C. albicans.*
- Presence of electron withdrawing group (–Br, Compound **11**) improved the antifungal activity of the synthesized compounds against *A. niger*.
- Using 5-bromo-2-hydroxybenzaldehyde (Compound **10**) improved the antibacterial activity of the synthesized compounds against Gram negative S*. enterica* and *P. aeruginosa*.
- NO_2_ group presence on benzylidene portion of acetophenone play an important role to enhanced the antimicrobial activity against bacterial and fungal microorganism.

### Experimental section

Starting materials were obtained from commercial sources and were used without any type of further purification. The completion of the chemical reaction was observed by thin layer chromatography (TLC) making use of silica gel G plates of 0.5 mm thickness as stationary phase and benzene as mobile phase for final compounds. Melting points of final compounds were determined by open capillary tubes method. The molecular structures of the compounds were characterized by ^1^H/^13^C-NMR (CDCl_3_, δ ppm), FT-IR and Mass spectral studies. The Mass spectral data were confirmed by Waters Micromass Q-ToF Micro instrument. ^1^H nuclear magnetic resonance (^1^H-NMR) spectra was recorded on Bruker Avance 400 MHz spectrometer in appropriate CDCl_3_ solvents and are expressed in parts per million (δ, ppm) downfield from tetramethyl silane (internal standard). ^1^H-NMR data are given as multiplicity (s, singlet; d, doublet; t, triplet; m, multiplet) and number of protons. Infrared (IR) spectra were recorded on Bruker 12060280, Software: OPUS 7.2.139.1294 spectrometer in the range of 400–4000 using KBr pellets and the value of λ max were reported in cm^−1^.

#### General procedure for synthesized pyrimidine analogues

##### Step i: synthesis of substituted chalcone (intermediate-I)

The reaction mixture of 1-(4-nitrophenyl)ethanone (0.01 mol) and corresponding aldehyde (0.01 mol) were stirred for 2–3 h in methanol (5–10 ml) followed by drop wise addition of sodium hydroxide solution (10 ml 40%) with constant stirring at room temperature. Then reaction mixture was taken overnight at room temperature and then was poured into ice cold water and acidified with hydrochloric acid and the precipitated substituted chalcone was filtered, dried and recrystallized from methanol [20].

##### Step ii: synthesis of 4-(substituted phenyl)-6-(4-nitrophenyl)pyrimidin-2-ol/thiol/amine derivatives

The solution of substituted chalcone (0.01 mol) [synthesized in “Step i: synthesis of substituted chalcone (intermediate-I)”] in methanol (50 ml) was added with 0.01 mol of potassium hydroxide and 40 ml of 0.25 M solution of thiourea/urea/guanidine hydrochloride and refluxed for 3–4 h. The reaction mixture was then cooled and acidified with few drops of hydrochloric acid (20 ml of 0.5 M solution) and the resultant precipitate 4-(substituted phenyl)-6-(4-nitrophenyl)pyrimidin-2-ol/thiol/amine was separated dried and recrystallized from methanol.

Spectral analysis determined by FT-IR (KBr pellets, cm^−1^) and ^1^H/^13^C-NMR (CDCl_3_, δ ppm).

##### 4-(2-Chlorophenyl)-6-(4-nitrophenyl)pyrimidin-2-amine (1)

M. Formula: C_16_H_11_ClN_4_O_2_; Yield: 75.00%; MS ES + (ToF): *m/z* 326 [M^+^ + 1]; IR (KBr pellets, cm^−1^): 2931 (C–H str.), 1596 (C=C str.), 700 (C–C str.), 1688 (C=N str. or N=CH str., pyrimidine ring), 1344 (C–N str., pyrimidine), 754 (C–Cl str.), 1521 (NO_2_ asym str.), 854 (C–N str., Ar–NO_2_), 3379 (NH_2_ asym str.); ^13^C-NMR (CDCl_3_-*d*_6_, δ, ppm): 163.4, 163.6, 160.1, 148.3, 139.8, 132.4, 130.1, 129.2, 128.3, 127.4, 121.7, 95.2; ^1^H-NMR (CDCl_3_, δ, ppm): 7.13–8.25 (m, 8H, Ar–H), 6.71 (s, 1H, CH of pyrimidine ring), 4.2 (s, 2H, NH_2_).

##### 4-(2-Chlorophenyl)-6-(4-nitrophenyl)pyrimidine-2-thiol (2)

M. Formula: C_16_H_10_ClN_3_O_2_S; Yield: 84.72%; MS ES + (ToF): *m/z* 343 [M^+^ + 1]; IR (KBr pellets, cm^−1^): 2858 (C–H str.), 1596 (C=C str.), 703 (C–C str.), 1665 (C=N str.), 1342 (C–N str., pyrimidine), 753 (C–Cl str.), 1521 (NO_2_ asym str.), 698 (C–N str., Ar–NO_2_), 2627 (S–H str.), 621 (C–S str.); ^13^C-NMR (CDCl_3_-*d*_6_, δ, ppm): 182.4, 163.5, 163.2, 160.1, 147.3, 139.6, 132.2, 130.1, 129.6, 128.3, 127.4, 121.7, 106.1; ^1^H-NMR (CDCl_3_, δ, ppm): 7.35–8.34 (m, 8H, Ar–H), 8.40 (s, 1H, CH of pyrimidine ring), 3.01(s, 1H, SH).

##### 4-(3-Chlorophenyl)-6-(4-nitrophenyl)pyrimidin-2-amine (3)

M. Formula: C_16_H_11_ClN_4_O_2_; Yield: 78.78%; MS ES + (ToF): *m/z* 326 [M^+^ + 1]; IR (KBr pellets, cm^−1^): 2923 (C–H str.), 1607 (C=C str.), 703 (C–C str.), 1670 (C=N str.), 1351 (C–N str., pyrimidine), 732 (C–Cl str.), 1525 (NO_2_ asym str., phenyl ring), 674 (C–N str., Ar–NO_2_), 3387 (NH_2_ asym str.); ^13^C-NMR (CDCl_3_-*d*_6_, δ, ppm): 163.2, 160.1, 147.2, 138.6, 132.0, 134.3, 130.1, 129.2, 128.1, 127.4, 125.3, 121.7, 95.3; ^1^H-NMR (CDCl_3_, δ, ppm): 7.26–9.02 (m, 8H, Ar–H), 6.0 (s, 1H, CH of pyrimidine ring), 4.3 (s, 2H, NH_2_).

##### 4-(3-Chlorophenyl)-6-(4-nitrophenyl)pyrimidine-2-thiol (4)

M. Formula: C_16_H_10_ClN_3_O_2_S; Yield: 72.54%; MS ES + (ToF): *m/z* 343 [M^+^ + 1]; IR (KBr pellets, cm^−1^): 2991 (C–H str.), 1570 (C=C str.), 709 (C–C str.), 1701 (C=N str. pyrimidine ring), 1303 (C–N str.), 748 (C–Cl str.), 1521 (NO_2_ asym str.), 659 (C–N str., Ar–NO_2_), 2597 (S–H str.), 709 (C–S str.); ^13^C-NMR (CDCl_3_-*d*_6_, δ, ppm): 181.4,163.5,163.2,160.1,146.3, 139.6, 132.2,130.1,129.6, 128.3,127.4, 125.3, 121.7, 103.1; ^1^H-NMR (CDCl_3_, δ, ppm): 7.83–8.25 (m, 8H, Ar–H), 7.41 (s, 1H, CH of pyrimidine ring), 3.06 (s, 1H, SH).

##### 4-(4-Chlorophenyl)-6-(4-nitrophenyl)pyrimidin-2-amine (5)

M. Formula: C_16_H_11_ClN_4_O_2_; Yield: 82.22%; MS ES + (ToF): *m/z* 326 [M^+^ + 1]; IR (KBr pellets, cm^−1^): 2942 (C–H str.), 1598 (C=C str.), 703 (C–C str.), 1673 (C=N str.), 1346 (C–N str., pyrimidine), 755 (C–Cl str.), 1523 (NO_2_ asym str.), 822 (C–N str., Ar–NO_2_), 3349 (NH_2_ asym str.); ^13^C-NMR (CDCl_3_-*d*_6_, δ, ppm): 162.2, 160.1, 146.2, 138.6, 131.0, 134.3 130.1, 129.2, 128.1, 127.4, 124.3, 121.7, 96.3; ^1^H-NMR (CDCl_3_, δ, ppm): 7.33–8.34 (m, 8H, Ar–H), 7.85 (s, 1H, CH of pyrimidine ring), 4.14 (s, 2H, NH_2_).

##### 4-(4-Chlorophenyl)-6-(4-nitrophenyl)pyrimidine-2-thiol (6)

M. Formula: C_16_H_10_ClN_3_O_2_S; Yield: 75.00%; MS ES + (ToF): *m/z* 343 [M^+^ + 1]; IR (KBr pellets, cm^−1^): 2927 (C–H str.), 1596 (C=C str.), 709 (C–C str.), 1345 (C–N str., pyrimidine), 824 (C–Cl str.), 1480 (NO_2_ asym str.), 698 (C–N str., Ar–NO_2_), 645 (C–S str.), 2602 (S–H str.); ^13^C-NMR (CDCl_3_-*d*_6_, δ, ppm): 182.4, 163.2, 163.2, 161.1, 148.3, 139.6, 131.2, 130.1, 129.6, 128.3, 126.4, 121.7, 103.4; ^1^H-NMR (CDCl_3_, δ, ppm): 7.83–8.25 (m, 8H, Ar–H), 7.45 (s, 1H, CH of pyrimidine ring), 3.34 (s, 1H, SH).

##### 4-(4-Chlorophenyl)-6-(4-nitrophenyl)pyrimidin-2-ol (7)

M. Formula: C_16_H_10_ClN_3_O_3_; Yield: 84.72%; MS ES + (ToF): *m/z* 327 [M^+^ + 1]; IR (KBr pellets, cm^−1^): 2941 (C–H str.), 1595 (C=C str.), 705 (C–C str.), 1672 (C=N str.), 1342 (C–N str., pyrimidine), 756 (C–Cl str.), 1523 (NO_2_ asym str.), 3374 (O–H str.), 822 (C–N str., Ar–NO_2_), 1404 (C–O str., and O–H in plane bending vib.); ^13^C-NMR (CDCl_3_-*d*_6_, δ, ppm): 160.4, 160.4, 153.2, 148.2, 139.1, 134.1, 131.1, 129.2, 128.2, 121.2, 88.1; ^1^H-NMR (CDCl_3_, δ, ppm): 7.43–8.56 (m, 8H, Ar–H), 6.61 (s, 1H, CH of pyrimidine ring), 5.04 (s, 1H, OH).

##### 4-(3-Methoxyphenyl)-6-(4-nitrophenyl)pyrimidin-2-amine (8)

M. Formula: C_17_H_14_N_4_O_3_; Yield: 73.43%; MS ES + (ToF): *m/z* 322 [M^+^ + 1]; IR (KBr pellets, cm^−1^): 2947 (C–H str.), 692 (C–C str.), 1709 (C=N str. pyrimidine ring), 1344 (C–N str., pyrimidine), 784 (C–N str., Ar–NO_2_), 1041 (C–O–C str., –OCH_3_), 2839 (C–H str., R–CH_3_); ^13^C-NMR (CDCl_3_-*d*_6_, δ, ppm): 163.2, 160.1, 146.2, 139.6, 131.0, 134.3 130.1, 128.1, 121.7, 119.3, 114.3, 111.3, 96.3, 55.2; ^1^H-NMR (CDCl_3_, δ, ppm): 6.33–8.44 (m, 8H, Ar–H), 6.85 (s, 1H, CH of pyrimidine ring), 4.2 (s, 2H, NH_2_), 3.34 (s, 1H, OCH_3_).

##### 4-(4-Methoxyphenyl)-6-(4-nitrophenyl)pyrimidin-2-amine (9)

M. Formula: C_17_H_14_N_4_O_3_; Yield: 76.47%; MS ES + (ToF): *m/z* 322 [M^+^ + 1]; IR (KBr pellets, cm^−1^): 2937 (C–H str.), 1604 (C=C str.), 694 (C–C str.), 1661 (C=N str.), 1349 (C–N str., pyrimidine), 1502 (NO_2_ asym str., phenyl ring), 752 (C–N str., Ar–NO_2_), 1108 (C–O–C str., –OCH_3_), 2842 (C–H str., R–CH_3_); ^13^C-NMR (CDCl_3_-*d*_6_, δ, ppm): 163.1, 160.1, 148.2, 139.6, 128.1, 125.3, 121.7, 114.3, 95.3; ^1^H-NMR (CDCl_3_, δ, ppm): 6.33–8.71 (m, 8H, Ar–H), 6.35 (s, 1H, CH of pyrimidine ring), 4.23 (s, 2H, NH_2_), 3.01 (s, 1H, OCH_3_).

##### 4-Bromo-2-(2-mercapto-6-(4-nitrophenyl)pyrimidin-4-yl)phenol (10)

M. Formula: C_16_H_10_BrN_3_O_3_S; Yield: 81.81%; MS ES + (ToF): *m/z* 404 [M^+^ + 1]; IR (KBr pellets, cm^−1^): 2869 (C–H str.), 1592 (C=C str.), 691 (C–C str.), 1680 (C=N str.), 1349 (C–N str., pyrimidine), 623 (C–Br str.), 1521 (NO_2_ asym str., phenyl ring), 844 (C–N str., Ar-NO_2_), 2597 (S–H str.), 623 (C–S str.); ^13^C-NMR (CDCl_3_-*d*_6_, δ, ppm): 182.4, 163.2, 161.1, 154.3, 148.3, 139.6, 134.2, 133.1, 128.3, 121.2, 122.4, 115.2, 118.2, 103.4; ^1^H-NMR (CDCl_3_, δ, ppm): 7.93–8.35 (m, 7H, Ar–H), 8.41 (s, 1H, CH of pyrimidine ring), 3.05 (s, 1H, SH), 5.97 (s, 1H, OH).

##### 4-(3-Bromophenyl)-6-(4-nitrophenyl)pyrimidin-2-amine (11)

M. Formula: C_16_H_11_BrN_4_O_2_; Yield: 64.00%; MS ES + (ToF): *m/z* 371 [M^+^ + 1]; IR (KBr pellets, cm^−1^): 3064 (C–H str.), 1596 (C=C str.), 692 (C–C str.), 1671 (C=N str.), 1342 (C–N str., pyrimidine), 1500 (NO_2_ asym str., phenyl ring), 783 (C–N str., Ar–NO_2_), 645 (C–Br str.); ^13^C-NMR (CDCl_3_-*d*_6_, δ, ppm): 163.2, 160.1, 148.2, 139.6, 131.0, 134.3, 130.1, 129.2, 128.1, 126.3, 121.7, 95.3; ^1^H-NMR (CDCl_3_, δ, ppm): 6.11–8.41 (m, 8H, Ar–H), 7.35 (s, 1H, CH of pyrimidine ring), 4.00 (s, 2H, NH_2_).

##### 4-(2,4-Dichlorophenyl)-6-(4-nitrophenyl)pyrimidin-2-amine (12)

M. Formula: C_16_H_10_ClN_4_O_2_; Yield: 87.61%; MS ES + (ToF): *m/z* 361 [M^+^ + 1]; IR (KBr pellets, cm^−1^):1600 (C=C str.), 695 (C–C str.), 1669 (C=N str.), 1346 (C–N str., pyrimidine), 848 (C–Cl str.), 1415 (NO_2_ asym str., phenyl ring), 735 (C–N str., Ar–NO_2_); ^1^H-NMR (CDCl_3_, δ, ppm): 6.34–8.67 (m, 7H, Ar–H), 6.15 (s, 1H, CH of pyrimidine ring), 4.30 (s, 2H, NH_2_); ^13^C-NMR (CDCl_3_-*d*_6_, δ, ppm): 163.6, 160.1, 147.2, 139.4, 133.3, 135.1, 129.2,128.1, 127.3, 121.7, 95.6.

##### 4-(4-(Dimethylamino)phenyl)-6-(4-nitrophenyl)pyrimidin-2-ol (13)

M. Formula: C_18_H_16_N_4_O_3_; Yield: 77.38%; MS ES + (ToF): *m/z* 336 [M^+^ + 1]; IR (KBr pellets, cm^−1^): 2923 (C*–*H str.), 1524 (C=C str.), 704 (C–C str.), 1670 (C=N str. or N=CH str., pyrimidine ring), 1348 (C–N str., phenyl ring), 733 (NO_2_ asym str., phenyl ring), 806 (C–N str., Ar. nitro group), 2858 (C–H str., R–CH_3_), 3393 (O–H str.); ^13^C-NMR (CDCl_3_-*d*_6_, δ, ppm): 160.5, 154.3, 149.2, 139.6, 128.1, 122.7,121.3, 114.3, 87.2, 41.1; ^1^H-NMR (CDCl_3_, δ, ppm): 6.11–8.26 (m, 8H, Ar–H), 6.75 (s, 1H, CH of pyrimidine ring), 5.30 (s, 1H, OH), 2.19 (s, 6H, (CH_3_)_2_).

## Conclusion

Summarizing, we may conclude that the synthesized compounds (**2**, **5, 10, 11** and **12**) displayed appreciable antibacterial and antifungal activities against Gram positive bacteria (*S. aureus* and *B. subtilis*), Gram negative bacteria (*E. coli, S. enterica* and *P. aeruginosa*) and fungal strains (*C. albicans* and *A. niger*). The electron withdrawing group’s play an important role to enhanced the antimicrobial potential of compounds **2**, **5, 11** and **12** and these compound more active than standard drugs cefadroxil and fluconazole. The MBC and MFC values represent the lowest concentration of compound that produces in the range of 96–98% end point reduction of the used test bacterial and fungal species.
